# Long-Term V-EEG in Epilepsy: Chronological Distribution of Recorded Events Focused on the Differential Diagnosis of Epileptic Seizures and Psychogenic Non-Epileptic Seizures

**DOI:** 10.3390/jcm10102080

**Published:** 2021-05-12

**Authors:** Fernando Vázquez-Sánchez, Beatriz García-López, Ana Isabel Gómez-Menéndez, Asunción Martín-Santidrián, Jesús Macarrón Vicente, Alicia Hernando-Asensio, Pedro Gámez-Beltrán, Jerónimo J. González-Bernal, Raúl Soto-Cámara, María Jiménez-Barrios, Josefa González-Santos

**Affiliations:** 1 Neurologist, Neurology Service, Burgos University Hospital, 09006 Burgos, Spain; fvazsan@saludcastillayleon.es (F.V.-S.); amartins@saludcastillayleon.es (A.M.-S.); jmacarron@saludcastillayleon.es (J.M.V.); ahernandoas@saludcastillayleon.es (A.H.-A.); pgamezb@saludcastillayleon.es (P.G.-B.); 2 Neurophysiologist, Neurophysiology Service, Burgos University Hospital, 09006 Burgos, Spain; bgarcialo@saludcastillayleon.es (B.G.-L.); agomm@saludcastillayleon.es (A.I.G.-M.); 3 Department of Health Sciences, University of Burgos, 09001 Burgos, Spain; mariajb@ubu.es (M.J.-B.); mjgonzalez@ubu.es (J.G.-S.)

**Keywords:** epileptic seizure (ES), psychogenic non-epileptic seizure (PNES), anti-epileptic drugs (AEDs), video-electroencephalogram and video-electroencephalography (V-EEG), electroencephalogram (EEG)

## Abstract

Differential diagnosis in epilepsy is sometimes challenging. Video-electroencephalography (V-EEG) is an essential tool in the diagnosis and management of epilepsy. The prolonged duration of V-EEG recording increases the diagnostic yield of a conventional V-EEG. The right length of monitoring for different indications is still to be established. We present a retrospective descriptive study with a sample of 50 patients with long-term V-EEG monitoring, with a mean age of 36.1 years, monitored from 2013 to 2019 at the Burgos University Hospital. The mean monitoring time was 3.6 days. Events were obtained in 76% of the patients, corresponding to epileptic seizures (ES) in 57.9% of them, with psychogenic non-epileptic seizures (PNES) in 39.5%, and with episodes of both pathologies in 2.6% of the patients. We found that the first event was highly representative, and it correlated with the rest of the events that would be recorded. Moreover, 92% of the first PNES had been captured at the end of the second day, and 89% of the first ES by the end of the third day. V-EEG for differential diagnosis between ES and PNES can be performed in hospitals without specialized epilepsy surgery units. For this indication, the duration of long-term V-EEG can be adjusted individually depending on the nature of the first event.

## 1. Introduction

The correct diagnosis of epilepsy has essential implications for the patient’s health, occupation, and social interactions. However, given the wide variety of events that resemble an epileptic seizure (ES), establishing a correct diagnosis is sometimes challenging [1].

Different studies show a general rate of misdiagnosis between 20% and 40%, which entails a series of negative psychological and socioeconomic repercussions for the patient and different economic implications for the health services [2,3,4].

Epilepsy and other paroxysmal events are diagnosed through both clinical presentation and V-EEG recordings. V-EEG is the recording of the bioelectrical brain activity coupled to a simultaneous video recording that can be thoroughly analyzed [5,6,7]. Its usefulness is not debated regarding any of its possible indications (diagnosis of epilepsy, differential diagnosis, presurgical workup, and treatment decisions) and modalities (standard scalp V-EEG, emergency V-EEG, ambulatory EEG monitoring, long-term V-EEG monitoring, and intracranial EEG monitoring) [8]. 

Recommendations for the use of long-term monitoring in epilepsy have been established by different scientific societies [9,10,11]. Nevertheless, considerable diversity in practice exists among centers depending on their resources, making the outcomes variable [12]. 

Non-epileptic psychogenic seizures (PNES) are known as episodes of paroxysmal deterioration of self-control that manifest as involuntary movements, sensations, or behavior. These episodes do not present by correlating the epileptiform electroencephalographic discharges or the clinical evidence of epilepsy; however, they can simulate some ES types, giving a poor control of events, and, therefore, they can lead to confusion between both. The coexistence between PNES and ES has been observed, making it necessary to consider the possibility of PNES, particularly in drug-resistant cases, without seizure control despite the correct antiepileptic drug (AED) treatment [13,14,15,16].

There is often a delay of years between the onset of PNES and its correct diagnosis. The ILAE considers that the latency until the diagnosis is about 3 years [17]. It produces uncertainty in the patient, worse results in their evolution, unnecessary use of AED, and other inappropriate interventions [18,19,20].

Some studies have shown an improvement in diagnostic accuracy when using long-term monitoring. The reported duration ranges between 24 and 144 h, making the result variable. Some authors concluded that the most efficient monitoring time was 4.8 days, with significant differences among studies. Other authors conclude that the duration may be shorter in cases with PNES, in whom they reach a diagnosis in the first 48 h, and a longer monitorization in patients with refractory epilepsy, with recordings of at least 72 h. However, the optimal monitoring time to obtain relevant results is unknown [15,21,22,23,24,25,26].

This study presents a cohort of patients who underwent long-term V-EEG with different indications at the Burgos University Hospital (HUBU). Its main objective is to evaluate the optimal monitoring duration under some specific indications by analyzing our series results. For this purpose, we focused on the time distribution of events, their variability throughout the recording, and the event’s etiology. Our secondary objective was to describe our population characteristics, as it may affect our results and their generalization.

## 2. Materials and Methods

### 2.1. Study Design—Participants 

We present a descriptive, retrospective study of a group of patients monitored at the HUBU. It is a third-level hospital that cares for patients with epilepsy, where there is the possibility of carrying out long-term V-EEG monitoring, mainly for differential diagnosis and improvement of the management of AED in pharmacoresistant epilepsy. Since epilepsy surgeries were not carried out in our center, candidates for a surgical approach were referred directly to surgical epilepsy center units, where the presurgical workup was achieved.

All patients who required long-term monitoring of more than 24 h for diagnostic purposes of their epilepsy were considered eligible for this study. The indication of monitoring was the need for an etiological diagnosis of events, a suboptimal seizure control in patients diagnosed with drug-resistant epilepsy, or the need for further information about their epileptogenesis in patients with epilepsy.

All patients were thoroughly studied by one or more conventional V-EEG and sleep V-EEG for up to 24 h as a previous workup. We excluded patients to whom this workup had contributed enough diagnostic information, and patients to whom this previous workup resulted in the need for a surgical evaluation.

### 2.2. Procedure—Main Outcomes

To date, the conventional duration of long-term V-EEG monitorization lasts up to four days in our center without making differences among indications and being longer if necessary to achieve the objective information for each case. Shorter monitoring has been recorded in some patients. When a candidate is considered eligible for monitoring in our weekly clinical session, we organize the activity to monitor the patient, usually on the last week of the month. We do not count on exclusively dedicated rooms for this purpose. However, we dispose of a room in the neurology department with a V-EEG Nihon Kohden 64 channels amplifier and a camera connected to the nursing control where there are trained nurses for the seizure evaluation. When there is no epilepsy monitorization, this room is used for other pathologies. 

During the V-EEG monitoring, the patient counts on the continuous presence of a companion. The patient has an alarm to warn when presenting with premonitory symptoms of any type of event and, if not, the companion is in charge of notifying the event. The recording is carried out through electrodes distributed according to the international 10/20 system [27], T1 and T2 as additional electrode recording positions, electrocardiogram, electrooculogram, chin electromyography, and respiratory polygraphy. There is also a neurologist on 24 h duty and a neurophysiologist available on call 24 h a day, 365 days a year in our hospital.

Intermittent photic stimulation, daily hyperventilation, and sleep deprivation are performed in all patients as activation measures and protocolized decrease in AED at a rate of one-third of the medication per day from the second day of hospitalization when required. According to the monitoring findings, the AEDs are individually modified to better tailor them to the patients’ clinical situation.

The evaluation of recorded clinical events and V-EEG is made every day by a neurologist and a neurophysiologist experienced in epilepsy. The neuroimaging performed in our center is evaluated by radiologists specialized in neuroradiology.

Patients included in the present study correspond to our 2013–2019 cohort. All of them had been referred for evaluation by general neurologists from our center and other hospitals in our region. A group of neurologists and neurophysiologists with experience in diagnosing and managing epilepsy evaluated the potential usefulness of a long-term V-EEG recording for every single case. 

Patients signed informed consent before the V-EEG monitorization explaining the purpose of the test, the video recording, and the possible risks associated with the activation measures, including the possibility of reducing AED. We designed a retrospective study for analyzing the correlation between the nature of the event and the temporal distribution of their appearance throughout the long-term V-EEG recording. This study was approved by the HUBU ethical committee registered under the number CEIC 2510.

### 2.3. Statistical Analysis

An anonymized database was created for the retrospective data analysis. We analyzed the time distribution of the first event and the temporal distribution of all of them according to their nature (ES or PNES). The non-parametric Mann–Whitney test was used to assess the correlation study of the timing distribution for each type of event since the variables did not meet the normality criteria.

Three of the analyzed patients recorded more than 30 events, which were coded as multiple. Only the first of the events was considered in the hourly distribution graphs to avoid interfering with the entire sample by considering them as “outliers”. Of these three patients, only two presented ES, and the remaining patient only presented PNES. Whole days were considered as 24 h after 12:00 (noon) of the first day, instead of natural days. 

## 3. Results 

We present a series of 50 patients monitored between 2013 and 2019, of which 26 were men and 24 women, with a mean age of 36.1 years (standard deviation (SD) ± 14.05). The mean monitoring time was 3.6 days (SD ± 0.83).

The indications of the recording were: to make a diagnosis of the nature of the events in 46% of the patients with their consequent differential diagnosis between ES and PNES; the evaluation of drug-resistant epilepsy in 32% of the patients; the assessment of the reason for a recent worsening of a previously controlled epilepsy in 18% of the patients; and to enhance the diagnostic information by improving the knowledge of the location of the origin of the epileptic discharge in 4%. 

**Figure 1:**
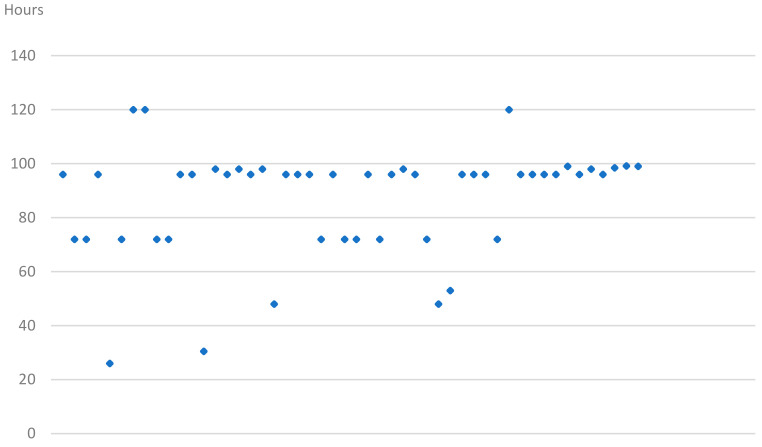
Duration of monitorization per patient in hours.

Our standard protocol was to admit patients for monitoring from Monday to Friday and initiating the recording at noon on Monday. The mean and median monitorization time in the series was 87.1 and 96 h, respectively (26–120) (Figure 1).

**Figure 2:**
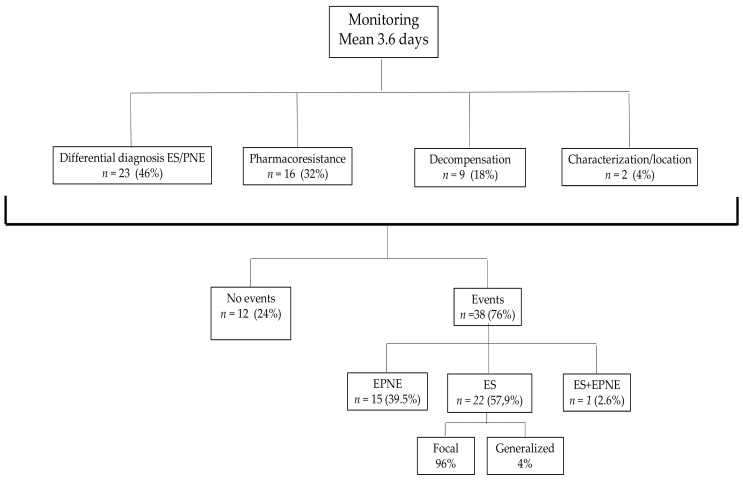
Flow chart describing the cohort.

Regarding the analysis of events, some of them were recorded in 38 of the monitored patients. We recorded a mean of 4.1 events per patient in the whole sample and a mean of 6.36 in patients with recorded events (SD = 9.03; median = 4). Almost all events could be labeled with various events classified as indeterminate in a single patient who also registered an ES. One patient presented status epilepticus during V-EEG monitoring. Of patients with events, 57.9% presented only ES, 39.5% PNES, and 2.6% both types. In the remaining 12 patients, no event was recorded despite having undergone AED reduction. We recorded PNES in 16 patients. According to Magaudda’s classification [28], they corresponded to hypermotor (18.75%), akinetic (25%), focal motor (31.25%), and subjective symptoms (25%). The event types were classified in the patients with registered events, accounting for focal epilepsy in96% of the cases and generalized epilepsy only in 4% (Figure 2). Of the patients with focal ES recorded, the temporal foci represented 36% of the total, frontal foci 24%, parietal 18%, multifocal 18%, and occipital foci 4%. 

**Figure 3:**
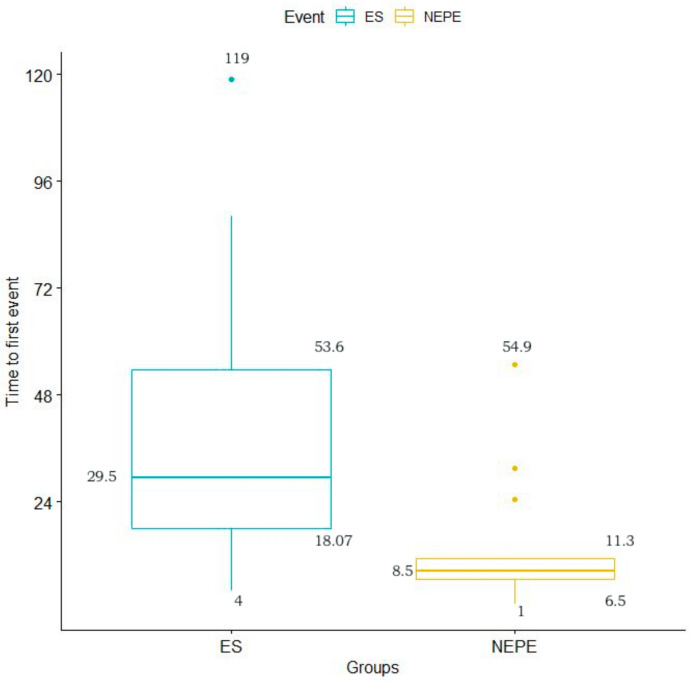
Average time of appearance of the first recorded event among the groups of patients.

We observed a shorter mean time in the PNES (mean = 14.02 h) than in the ES (mean = 39.55 h) when comparing the time of appearance of the first event recorded. This difference reached a statistical significance (*p* = 0.009) (Figure 3).

**Figure 4:**
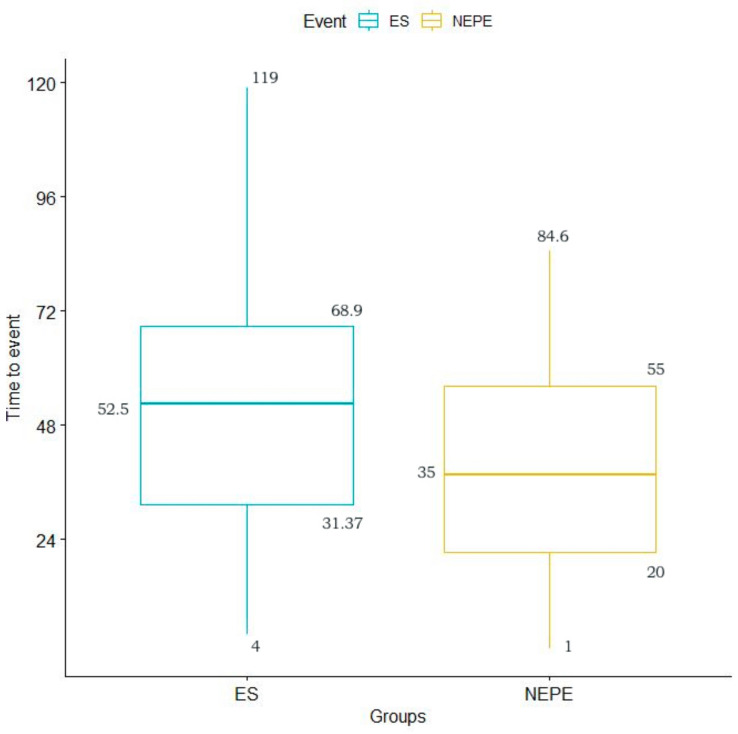
Average time of appearance of the total recorded events for PNES and ES.

Similarly, we observed a statistically significant difference in the temporal distribution of the total events: PNES appeared earlier (mean = 38.95 h or 1.5 days) than ES (mean = 51.72 h or 2.16 days) (*p* = 0.007) (Figure 4).

**Figure 5:**
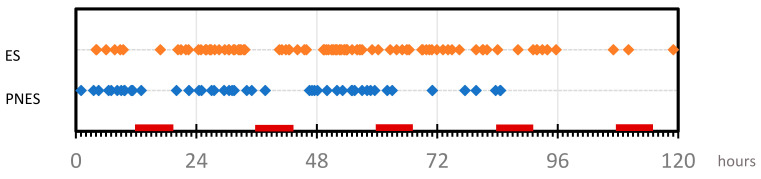
Temporal distribution of the events recorded during the monitoring time. Red marks in time-line indicate night time (from midnight to 7 a.m.).

In the V-EEG recording reports, a total of 168 events were characterized. The temporality of the events shows an early appearance of PNES, with the increase in the amount of ES after 24 h coinciding with the first reduction in AED, and this highlighted that PNES respect the hours of night rest, while ES appears at any time (Figure 5).

**Figure 6:**
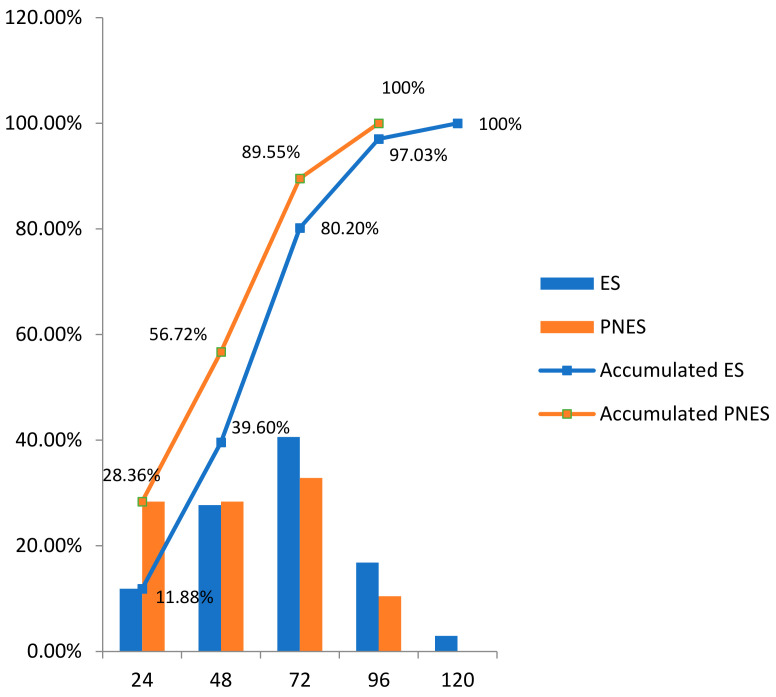
Distribution of daily accumulated events.

Concerning the distribution of all recorded events, at the end of the third day (72 h), 89.55% of PNES and 80.20% of ES were recorded (Figure 6).

**Figure 7:**
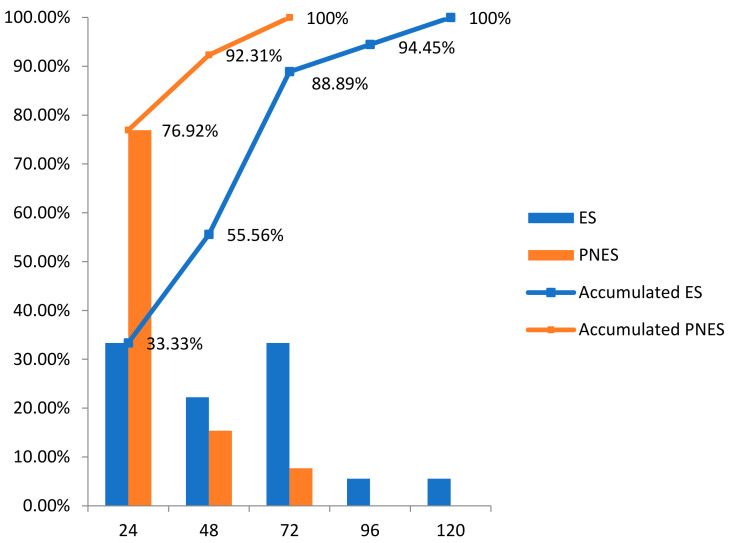
Distribution of the first event.

Regarding the first event’s distribution in patients with PNES, the first event was recorded in the first 24 h in 76.9%, reaching 92.31% in the first 48 h, while among patients with ES, 88.9% had presented their first event by the end of the third day (Figure 7). A total of 55% of all first events, despite their nature, were recorded within the first 24 h of monitoring. 

Of the 38 patients with registered events, 76.31% (*n* = 29) had been referred by only one type of stereotyped event phenotype, and 23.6% (*n* = 9) presented different types of events clinically.

In patients with clinically one type of spell, the first recorded event was superimposable to the ones that motivated the referral in 97.3% of the patients. Four of these patients only presented one recorded event, and the rest (*n* = 25) presented two or more recorded events. In 96% of patients with several events recorded, the first one had the same clinical and EEG features as the rest of them.

Nine out of the 38 patients had presented different clinical spells before monitoring. Their first recorded event was coincident with one type of their clinical spells in 88.9% of these patients. When several spells were recorded (70%, *n* = 6), they were superimposable to the first recorded event in 66.6% (*n* = 4) and different in the rest (*n* = 2).

Analyzing the concordance of the nature of the first event with the final diagnosis of the spells’ etiology, we found a predictive value of 92%. There were three exceptions: one case with several types of spells in his medical records and only one event recorded; one patient with both PNES and ES recorded; and one case with several recorded events, the first being to brief and impossible to classify.

## 4. Discussion

PNES is one of the most common spells misdiagnosed with ES, often leading to a wrong diagnosis, unnecessary AED treatment, and even more aggressive therapeutic measures [29]. In the case of PNES, its signs are determined mainly through the objective observation of clinical episodes, achieving diagnostic certainty during V-EEG recording. Due to the possible variability of spells, the optimal monitoring duration is under debate [17,26,30]. Looking for a universal answer for all indications of V-EEG monitoring and type of spell may not be realistic, but several precise data may help better adapt the timing of monitoring for some indications. 

An early diagnosis of PNES is essential to avoid unnecessary therapies, and it has been related to a better outcome at follow-up [31]. Even authors that have only found a trend in this relationship without a statistical significance recognize the interest in making the diagnosis as soon as possible, as an acceptance of diagnosis by the patient is essential for reducing episodes [32,33].

Epilepsy units with V-EEG recording for every indication, including presurgical evaluation, tend to have long delays in performing the V-EEG recording. Carrying out these studies in centers with similar characteristics to ours can improve the outcome by reducing the time until diagnosis, avoiding long-lasting wrong diagnosis, and reducing patients’ referral without epilepsy to specialized epilepsy units [34].

Our series patients are smaller than in other reports as we do not record presurgical candidates. 

The global yield in the number of patients with recorded events varies among series. It is reasonable to think that it depends on the characteristics of selected patients and the selection criteria to perform long-term monitoring. The inclusion of surgical patients increases the global amount of registered seizures due to more severe epilepsy. Alving et al. reported a thorough selection of all patients before the long-term monitoring, performing an extensive workup including repeated awake and sleep V-EEG, selecting only the “most difficult” cases for long-term monitoring [34]. Other factors such as the duration of EEG recordings and the provocation maneuvers used may play a role. Villanueva et al. reported a global yield of 97% of patients with registered events [35], while Foong et al. and Woollacott et al. reported 52.2% and 52.7%, respectively. In the three series, patients in a workup for epilepsy surgery were included [36,37]. We recorded events in 76% of patients in our cohort. Zanzmera et al. conducted a study with 1197 patients monitored for all V-EEG indications. They found short-term monitoring to be extremely useful, especially for PNES diagnosis, in which 80.7% of the patients with a final diagnosis of PNES did not require long-term monitoring. Their short-duration EEG ranged from 40 min to 120 min without specifying the length of the long duration V-EEG. In their study, spontaneous PNES were obtained in 64.9% of the patients with short-term monitoring and an additional 24.5% using activation techniques, such as verbal and tactile suggestion. They needed long-term monitoring for the rest of the patients [38]. Alving et al. only found 7% of PNES in their long-term monitoring series, as they had recorded most PNES in the extensive pre-long-term monitoring V-EEG workup performed. Their long-term monitoring duration was 2.35 days (55 h) for non-surgical indication [34]. We used hyperventilation, photic stimulation, and sleep deprivation in all of our patients as activation techniques. We also reduced AEDs in 76% of the total patients from the second day of monitoring. Nevertheless, we did not use any suggestions or placebo measures as an activation technique as other authors previously reported due to ethical concerns [39].

Regarding the etiological distribution of the events, in the study carried out by Lobello et al., with a total of 199 patients monitored for an average of 3 days, a series was presented to make a differential diagnosis between ES and PNES, where 45% of PNES were obtained compared to only 19% of ES [40]. The opposite occurred in the study by Villanueva et al., where presurgical patients were included, and the ES rate was higher, affecting 87% versus 13% for PNES [34]. We found 57.9% of ES and 39.5% of PNES.

We registered 16 patients with PNES (one of them also having ES) and classified them according to Magaudda [28]. We think that the low percentage of hypermotor PNES in our series (18.75%) could be related to the easier identification of this event as PNES through the clinical observation and the V-EEG workup prior to the long-term monitoring. Seneviratne et al. reported a high yield of correct classification of motor events into PNES and SE only by clinical observation by general neurologists [41]. Patients with subjective symptoms (25%) constituted, in our series, the group with the most challenging clinical presentation in the differential diagnosis between PNES and ES. Concerning registered events’ temporal distribution, more than 90% of the events had been recorded by the end of the fourth day, according to different studies. No differences were found between hospitalizations greater than or equal to 5 days and less than or equal to 4 days concerning the diagnostic conclusion [36,40,42]. In the study by Foong et al., in which monitoring was carried out with 207 patients for an average of 3.46 days, a low diagnostic yield was observed from the fifth day since 96.3% of the total events were registered in the first four days [36]. Our monitoring time was similar to the last series, as we had a mean monitoring time of 3.6 days with a median of 96 h. We recorded 89.6% of all PNES and 80.2% of all ES in the first three days of monitoring. We recorded 48.16% of the total events at the end of the first 48 h of monitoring. Patients with both PNES and ES are the most challenging for etiologic diagnosis. They present with different clinical and not always “constant pattern” events. We found only 2.6% of patients in our series, but other authors describe up to 7.3% in the same recording [43]. 

For patients with PNES, we recorded 100% of their first seizure at the end of the third day, with 76.92% in the first 24 h. In patients with ES, we recorded nearly 95% of their first event at the end of the fourth day, with 88.89% at the end of the third day.

Moreover, Foong et al. captured 50% of the patients with events by recording at least one event on the first day of monitoring regardless of the seizure type [36]. We captured 55% of the first event regardless of the event’s nature in the first 24 h. Regarding the timing of capturing events, in the monitoring carried out by Woollacott et al. with 254 patients, including presurgical workup studies, 15.72% of the PNES events occurred before or during the electrode placement, and no patient with ES had events in that period. In 98.5% of the PNES and 100% of the ES, the first event occurred within 48 h after the electrodes’ placement [37]. In the other series analyzed, ES was observed before PNES but without significant differences between both [35,36]. These differences might be related to a different population of the study, as more severe epilepsy probably has more episodes, and they could appear sooner throughout the monitorization. However, in the study by Rose et al., which was carried out with a series of 514 patients, statistically significant differences were observed between the time of appearance of the first ES with a mean of 2.1 days versus 1.2 days in the case of PNES [44]. These results coincide with those obtained in the present study since the mean of the time of appearance of the first clinical event was shorter in PNES (14.02 h) than in the case of ES (39.55 h), with statistically significant differences (*p* = 0.009). 

We found that the first event was highly representative, and it correlated with the rest of the events that would be recorded. We obtained 97.3% of coincidence in patients with just one type of pattern spell in their clinical history and 66.6% of coincidence in patients with several types of spells. The accuracy of the first event to predict the nature of the final diagnosis was 92%. This fact means that once the first event is recorded, one might review the monitoring aim to discuss the interest in individually adapting the length recording. This finding is especially significant in patients with only one type of event in their clinical history.

Once the monitoring results were studied, a reclassification of the type of epilepsy could be accurately made. Of the patients with epilepsy, 96% had focal epilepsy, and the percentage of generalized epilepsy was only 4% of the patients. This percentage is lower than in the series of Villanueva and Foong, who found 14.9% and 15.4%, respectively [33,36]. 

The study findings must be considered within the context of their strengths and limitations. Among their strengths is the integrity of the data collected due to our protocolized recording of medical history and systematic description of events recorded. Excluding patients with an indication for presurgical evaluation contributes to greater relevance to the findings obtained on the differential diagnosis between ES and PNES. Regarding limitations, this is a retrospective study carried out in a single center with a reduced sample of 50 patients, and all of them in adulthood. The lack of patients for surgery evaluation can also be a limitation since it could avoid the generalization of its conclusions to the series including presurgical monitoring. Including only patients with more than 24 h of monitoring may have provoked a selection bias. Nevertheless, these results could be valuable for recording a series similar to ours.

## 5. Conclusions

A gold standard for the duration of long-term V-EEG monitoring does not seem to exist. Its length should be adapted according to the information required, the information obtained throughout the register, the center resources, and the study population. 

The information obtained during the monitoring can be helpful to decide the duration of the long-term V-EEG, taking into account that the first recorded spell is highly representative of the rest of the events and the final diagnosis and that PNES appears significantly sooner than ES (mean of 14.02 h versus 39.55 h for the first event recorded).

Once the first event is recorded, one might review the monitoring aim to discuss the interest in individually adapting the length recording. We recorded 55% of the first events despite their nature in the first 24 h and 84.75% of all recorded events in the first 72 h. These data can also guide the total duration of the monitoring.

Long-term V-EEG monitoring in centers without surgical epilepsy units may be helpful to reduce referral time for epileptic patients to reach epilepsy specialized surgical units. At the same time, making an early diagnosis and correct management of PNES improve its outcome and prevent patients without epilepsy from being referred to specialized surgical centers.
